# Supplementary material for: Investigating the Prediction Accuracy of Recently Updated Intraocular Lens Power Formulas with Artificial Intelligence for High Myopia
Source: J Clin Med. 2022 Aug 18;11(16):4848. doi: 10.3390/jcm11164848 (PMC9410068; doi:10.3390/jcm11164848)
Supplement: Supplementary file 1 [file jcm-11-04848-s001.zip › jcm-1789783-supplementary.pdf]

Supplemental Table S1. *p* Values in Post Hoc Analyses.

	Hill vs. Kane	Hill vs. BUII	Hill vs. Haigis	Hill vs. SRK/T	Kane vs. BUII	Kane vs. Haigis	Kane vs. SRK/T	BUII vs. Haigis	BUII vs. SRK/T	Haigis vs. SRK/T
Prediction error	1.0	<0.001 *	<0.001 *	<0.001 *	<0.001 *	<0.001 *	<0.001 *	<0.001 *	<0.001 *	<0.001 *
Absolute error	1.0	<0.001 *	0.85	1.0	<0.001 *	0.83	1.0	1.0	1.0	1.0
Within ±0.25 D	1.0	0.014 *	0.4	1.0	0.34	1.0	1.0	1.0	1.0	1.0
Within ±0.50 D	N/A	N/A	N/A	N/A	N/A	N/A	N/A	N/A	N/A	N/A
Within ±1.00 D	N/A	N/A	N/A	N/A	N/A	N/A	N/A	N/A	N/A	N/A

* $p < 0.05$. Hill = Hill-RBF3.0; BUII = Barrett Universal II; D = diopters. Wang Koch adjustment was applied for the Haigis and SRK/T formulas.

Supplemental Table S2. *p* Values in Post Hoc Analyses in Eyes with Axial Length > 28.0 mm.

	Hill vs. Kane	Hill vs. BUII	Hill vs. Haigis	Hill vs. SRK/T	Kane vs. BUII	Kane vs. Haigis	Kane vs. SRK/T	BUII vs. Haigis	BUII vs. SRK/T	Haigis vs. SRK/T
Prediction error	0.0025 *	<0.001 *	<0.001 *	<0.001 *	<0.001 *	<0.001 *	<0.001 *	<0.001 *	<0.001 *	0.003 *
Absolute error	0.76	0.17	0.015 *	0.21	0.32	0.067	0.69	1.0	1.0	0.035 *
Within ±0.25 D	0.74	0.43	0.022 *	0.96	1.0	0.26	1.0	1.0	1.0	0.46
Within ±0.50 D	1.0	1.0	0.7	1.0	0.41	0.16	0.46	1.0	1.0	1.0
Within ±1.00 D	1.0	1.0	1.0	1.0	1.0	1.0	1.0	1.0	1.0	1.0

* $p < 0.05$. Hill = Hill-RBF3.0; BUII = Barrett Universal II; N/A = not applicable; D = diopters. Wang Koch adjustment was applied for the Haigis and SRK/T formulas.

Supplemental Table S3. *p* Values in Post Hoc Analyses in Eyes with Axial Length < 28.0 mm.

	Hill vs. Kane	Hill vs. BUII	Hill vs. Haigis	Hill vs. SRK/T	Kane vs. BUII	Kane vs. Haigis	Kane vs. SRK/T	BUII vs. Haigis	BUII vs. SRK/T	Haigis vs. SRK/T
Prediction error	1.0	<0.001 *	<0.001 *	<0.001 *	<0.001 *	<0.001 *	<0.001 *	<0.001 *	<0.001 *	0.0014 *
Absolute error	1.0	0.0024 *	1.0	1.0	0.0019*	1.0	1.0	1.0	0.92	1.0
Within ±0.25 D	N/A	N/A	N/A	N/A	N/A	N/A	N/A	N/A	N/A	N/A
Within ±0.50 D	N/A	N/A	N/A	N/A	N/A	N/A	N/A	N/A	N/A	N/A
Within ±1.00 D	N/A	N/A	N/A	N/A	N/A	N/A	N/A	N/A	N/A	N/A

* $p < 0.05$. Hill = Hill-RBF3.0; BUII = Barrett Universal II; N/A = not applicable; D = diopters. Wang Koch adjustment was applied for the Haigis and SRK/T formulas.
